# Spin–valley protected Kramers pair in bilayer graphene

**DOI:** 10.1038/s41565-025-01858-8

**Published:** 2025-02-10

**Authors:** Artem O. Denisov, Veronika Reckova, Solenn Cances, Max J. Ruckriegel, Michele Masseroni, Christoph Adam, Chuyao Tong, Jonas D. Gerber, Wei Wister Huang, Kenji Watanabe, Takashi Taniguchi, Thomas Ihn, Klaus Ensslin, Hadrien Duprez

**Affiliations:** 1 Laboratory for Solid State Physics, ETH Zurich, Zurich, Switzerland; 2 Research Center for Functional Materials, National Institute for Materials Science, Tsukuba, Japan; 3 International Center for Materials Nanoarchitectonics, National Institute for Materials Science, Tsukuba, Japan; 4 Quantum Center, ETH Zürich, Zürich, Switzerland

**Keywords:** Electronic properties and materials, Electronic properties and devices, Qubits, Quantum dots, Two-dimensional materials

## Abstract

The intrinsic valley degree of freedom makes bilayer graphene (BLG) a unique platform for semiconductor qubits. The single-carrier quantum dot (QD) ground state exhibits a twofold degeneracy, where the two states that constitute a Kramers pair have opposite spin and valley quantum numbers. Because of the valley-dependent Berry curvature, an out-of-plane magnetic field breaks the time-reversal symmetry of this ground state and a qubit can be encoded in the spin–valley subspace. The Kramers states are protected against known spin- and valley-mixing mechanisms because mixing requires a simultaneous change of the two quantum numbers. Here, we fabricate a tunable QD device in Bernal BLG and measure a spin–valley relaxation time for the Kramers states of 38 s at 30 mK, which is two orders of magnitude longer than the 0.4 s measured for purely spin-blocked states. We also show that the intrinsic Kane–Mele spin–orbit splitting enables a Kramers doublet single-shot readout even at zero magnetic field with a fidelity above 99%. If these long-lived Kramers states also possess long coherence times and can be effectively manipulated, electrostatically defined QDs in BLG may serve as long-lived semiconductor qubits, extending beyond the spin qubit paradigm.

## Abstract (web summary)

A tunable quantum dot device in Bernal bilayer graphene possesses a spin–valley relaxation time of 38 s at millikelvin temperatures.

## Main

Atomically thin semiconductors with a hexagonal crystal lattice serve as a unique host for solid-state qubits^1,2^ due to their intrinsic valley degree of freedom. Among them, bilayer graphene (BLG) stands out as a platform that benefits from carbon’s low nuclear spin density and weak spin–orbit coupling, along with the ability to fabricate highly tunable few-carrier quantum dots (QDs)^3–5^. The twofold valley degeneracy^6,7^ provides an opportunity to define a qubit in the two-dimensional subspace spanned by the valley and the spin degrees of freedom^8^. One benefit of encoding a qubit using two quantum numbers instead of one is its robustness (protection) against different types of noise that would otherwise cause it to decay. While the spin is coupled to surrounding magnetic moments and phonons via spin–orbit coupling^9^ or hyperfine interaction^10^, a valley flip requires a scattering process with a giant momentum swing of the order of the length of the reciprocal lattice vector, which is only known to be caused by short-range perturbations on the length scale of atomic defects. Given the low disorder reached in encapsulated graphene^11^, the valley relaxation times were demonstrated to be at least an order of magnitude longer than spin relaxation times^3^ within a single device. We can thus speculate that the simultaneous flip of both spin and valley^12^ is a rare second-order process.

A remarkable property of BLG is a large out-of-plane valley *g* factor (*g*_v_), which originates from the finite Berry curvature around the K points^13^ and can be tuned using the electric field across a wide range of values from 10 to 100 (ref. ^14^). At zero magnetic field, the fourfold single-particle degeneracy is already lifted by Kane–Mele spin–orbit interaction^15^. It separates the energy of the two Kramers doublets (|K^−^↑〉, |K^+^↓〉) and (|K^−^↓〉, |K^+^↑〉) by a spin–orbit gap, which has been previously measured indirectly^16–18^ and directly^17,19^ to be *Δ*_SO_ ≈ 40–80 μeV $\widehat{=}$ 10–20 GHz. Comparable spin–orbit splittings are observed between Kramers pairs in semiconducting transition metal dichalcogenide multilayer systems, such as MoS_2_^12^. On increasing out-of-plane magnetic field *B*_⊥_, the time-reversal symmetry is broken, resulting in an energy splitting of both Kramers pairs. Previous studies have shown coherent oscillations of a spin–valley qubit formed in a carbon nanotube^20^ but yielded short relaxation times *T*_1_ < 10 μs (refs. ^20,21^) and decoherence times ${T}_{2}^{{\rm{(echo)}}}=65\,{\rm{ns}}$, presumably attributable to high levels of static disorder and charge noise^20–22^. In contrast, encapsulated BLG is known to be atomically flat, and with notably lower valley-mixing rate ${\Delta }_{{{\rm{K}}}^{+}{{\rm{K}}}^{-}} < 2\,{\rm{neV}}$ (ref. ^3^), promising longer relaxation times.

In this work, we perform relaxation time measurements of a single-hole QD formed in BLG with small (~mT) or zero applied magnetic field. We find *T*_1_ for simultaneous spin–valley relaxation to be as long as ${T}_{1}^{({\rm{sv}})}\approx 30\,{\rm{s}}$, which is two orders of magnitude longer than for pure spin relaxation, ${T}_{1}^{({\rm{s}})}\approx 0.4\,{\rm{s}}$. Additionally, we demonstrate high-fidelity readout at zero magnetic field where relaxation happens between two Kramer’s doublets and requires either a spin or a valley flip.

**Fig. 1:**
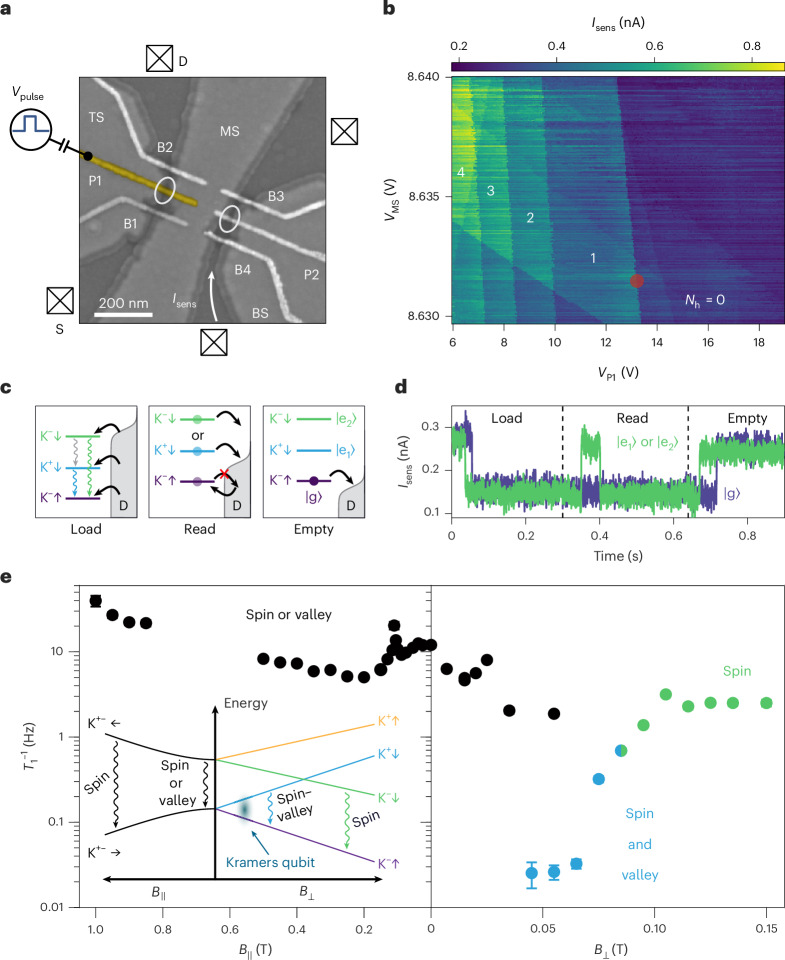
Device and pulsing protocol used to determine relaxation times. **a**, Scanning electron microscopy false-colour image of the device. All labelled metallic gates, including barrier gates (B1–B4), plunger gates (P1, P2) and split gates (TS, MS, BS), and the graphite back gate (not shown), as well as the source (S) and drain (D) ohmic contacts, are direct current (DC) biased. P1 is additionally controlled by alternating current pulses. **b**, Charge stability diagram of the dot probed with a charge sensor current *I*_sens_ as a function of *V*_MS_ and *V*_P1_. *N*_h_ indicates the number of hole carriers inside the QD. The red circle indicates the point on the gate–gate diagram where all measurements were performed. **c**, Three-step pulse readout protocol. Schematic energy diagrams for the three lowest states of the single hole in the BLG QD at *B*_⊥_ = 40 mT. **d**, Single-shot readout of the hole state. Typical time traces of *I*_sens_ in response to a three-level pulse. For the green trace, the hole is declared to be in one of the ESs according to the characteristic current step during the Read phase. For the purple trace, the hole is declared to be in the GS if no step is observed during the Read phase. **e**, Measured inverse relaxation time *T*_1_^−1^ as a function of *B*_∥_ and *B*_⊥_. Circles of different colours at the same magnetic field correspond to different relaxation channels controlled by changing the amplitude of Load and Read pulses. Data points are presented as mean values ± the s.d. of the calculated *T*_1_ (Methods). Some error bars are smaller than the symbol size of the data point. The inset shows the energy spectrum of a single carrier in the BLG QD plotted as a function of in-plane and out-of-plane magnetic fields.

We form a tunable QD device, electrostatically defined in Bernal BLG, using two layers of overlapping gates depicted in Fig. 1a (see Methods for fabrication details). The lower gate layer serves the purpose of confining the BLG charge carriers to two conducting channels via opening the band gap in BLG underneath the top (TS), middle (MS) and bottom (BS) split gates, and placing the Fermi energy in the gap in these regions^23^. The upper gate layer of narrow finger gates is used to precisely define the QDs within the channels. We form the signal and the sensor QDs underneath their respective plunger gates P1 and P2. The dot-to-reservoir couplings can be tuned individually by pairs of barrier gates (B1, B2) and (B3, B4). We tune the dot into the single-hole regime as shown in Fig. 1b, where the sensor current *I*_sens_ is shown as a function of middle split and plunger gate voltages *V*_MS_ and *V*_P1_. From the width of the 0–1 charge transition, we determine the electron temperature to be *T*_e_ ≈ 30 mK.

## Three-level single-shot readout

We perform the standard Elzerman three-level single-shot readout^4,24,25^ to extract the relaxation times (see Methods for the details). We apply voltage pulses to P1, while monitoring the real-time charge occupancy of the dot using the charge sensor. The concept of the readout is sketched in Fig. 1c: if a hole remains in the excited state (ES) after the ‘Load’ and during the ‘Read’ phase without relaxing, then, at some random time governed by the tunnelling-out rate, it will tunnel to the leads and thereafter the ground state (GS) of the dot will be occupied again through a tunnelling-in process. This in-and-out tunnelling manifests itself as a step in the sensor current shown in Fig. 1d (green trace). By determining the ratio of the number of shots with a single step to the total number of successfully loaded shots and discarding error traces (see Methods for postselection procedure), we extract the relaxation time by fitting the exponential decay as a function of the load time^26^.

Figure 1e shows the main results of this paper: the measured *T*_1_ are plotted as a function of small out-of-plane and in-plane magnetic fields. As we will prove later by ES spectroscopy measurements, at *B*_⊥_ < 80 mT the transition between the two lowest energy states in the single-particle spectrum are spin–valley blocked, so the resulting *T*_1_ times can be interpreted as spin–valley relaxation times ${T}_{1}^{\,{\rm{(sv)}}}$ (blue points in the figure). We observed long relaxation times ${T}_{1}^{\,({\rm{sv}})}\approx 30\,{\rm{s}}$. This is in tune with the intuition that a simultaneous flip of the two quantum numbers is a very rare second-order relaxation process. Note that the range in magnetic field where we can measure this spin–valley relaxation time is bounded from below by *B*_⊥_ ≳ 40 mT and from above by *B*_⊥_ ≲ 70 mT. We notice that at lower energy splittings the fidelity of Elzerman readout markedly drops, affected by finite temperature and charge noise^27^. We would like to note that similarly long *T*_1_ times were measured in GaAs^10^ in the limit close to *B* = 0. In both cases the times are probably limited by the required measurement time and sample stability rather than by the two-level system itself.

At low perpendicular magnetic fields, by choosing the Read level such that the Fermi energy *E*_F_ in the lead is inside the gap between the Kramers pairs, we can perform high-fidelity readout of the pair state and measure relaxation times between two doublets that are blocked by either spin or valley flip (black points in Fig. 1e). As a result, the resulting relaxation time is dominated by the fastest of two channels and can be expressed as ${T}_{1}={(1/{T}_{1}^{\,({\rm{s}})}+1/{T}_{1}^{\,({\rm{v}})})}^{-1}$.

Applying a perpendicular magnetic field lifts the Kramers pairs’ degeneracies, and enables us to distinguish the spin and spin–valley relaxation rates by accurately placing the *E*_F_ of the lead between the corresponding states. Around *B*_⊥_ ≈ 80 mT in Fig. 1e, two ESs cross, namely |K^−^↓〉 and |K^+^↓〉. For *B*_⊥_ > 80 mT, the transition between the two lowest states, |K^−^↓〉 and |K^−^↑〉, is purely spin blocked, meaning that the measured *T*_1_ times can be identified with spin relaxation times ${T}_{1}^{\,({\rm{s}})}$ (green data points). We measured ${T}_{1}^{\,({\rm{s}})}\approx 0.4\,{\rm{s}}$, which is two orders of magnitude shorter than the spin–valley relaxation time, but still one order of magnitude longer than spin relaxation times previously measured at higher magnetic fields *B*_⊥_ > 1.5 T (refs. ^3,4^). This finding is consistent with phonon spin relaxation mediated by spin–orbit coupling and/or hyperfine coupling^10,28^. Such long spin *T*_1_ times are also consistent with the fact the QD is highly decoupled from the leads with the intrinsic tunnelling rate as low as *Γ*_out_ ≈ 15 Hz (see Methods for *Γ*_in/out_ extraction). Additionally, we find no relaxation hotspot^9,29^ where two different valley states cross (at *B*_⊥_ = 80 mT) in Fig. 1e. This is consistent with the low intervalley mixing term measured in BLG^3,19^. In Methods, we explicitly rule out the argument that the observed long spin–valley relaxation times could be interpreted as fully dominated by pure spin relaxation.

In Fig. 1e(left) we also show the non-monotonic dependence of *T*_1_ on in-plane magnetic field *B*_∥_, which does not break the Kramers degeneracy but slowly increases the energy splitting of the Kramers pairs. Starting from *T*_1_ ≈ 100 ms at zero magnetic field, the relaxation time doubles around *B*_∥_ = 200 mT and then decreases monotonically at higher fields, reaching *T*_1_ ≈ 25 ms at *B*_∥_ = 1 T. We also observe two insignificant relaxation hotspots around *B*_∥_ = 110 mT and *B*_⊥_ = 20 mT. These hotspots leave no traces in spectroscopy measurements (Supplementary Fig. 2).

## Single-shot ES spectroscopy

**Fig. 2:**
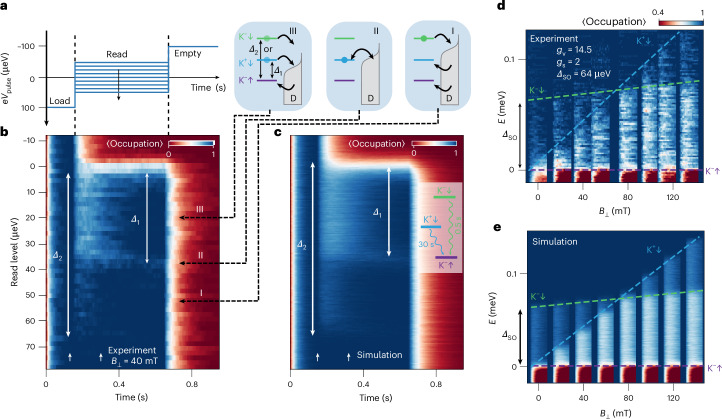
Single-shot energy spectroscopy. **a**, Calibration of the Read level and the ES spectroscopy. The Read level is varied while the Load and Empty are kept constant. **b**, The digitized sensor current (representing dot occupation) averaged over ~140 single-shot traces as a function of the Read level shift relative to the GS at *B*_⊥_ = 40 mT. Insets: the relative positions of the dot’s energy levels and the *E*_F_ of the lead for three different regimes: (III) readout from both ESs, (II) resonant jumps between the lower-energy ES and the lead and (I) readout from the higher-energy ES. *Δ*_1_ and *Δ*_2_ denote the energy splittings between the GS and the two ESs respectively. The sensor signal is digitized to represent the dot occupation, such that current offsets due to the sensor drift are compensated. **c**, Monte Carlo simulation of the single-shot readout averaged over 10,000 shots for each Read level. We use the simplest model with three states, as shown in the sketch, where both of the ESs can only relax to the GS with ${T}_{1}^{\,({\rm{s}})}=0.5\,{\rm{s}}$ and ${T}_{1}^{\,({\rm{sv}})}=30\,{\rm{s}}$—two values measured independently. **d**, ES spectroscopy. The beginning of the Read phase (spanning the time window marked with white arrows in **b**) is shown for *B*_⊥_ = [0, 20, 40, 55, 80, 100, 115, 135] mT. The Read level shift relative to the GS. The dashed lines show the evolution of the two lower-energy ESs in a single-hole BLG QD: |K^−^↓〉 and |K^+^↓〉, with *Δ*_SO_ = 64 μeV and *g*_v_ = 14.5. **e**, Data similar to those in **d** but for Monte Carlo simulated single-shot readout of the QD. The averaged step maps do not reveal solid evidence for the existence of the fourth state |K^+^↑〉, presumably due to the short relaxation time of the hole in this state. However, we could observe all four non-degenerate states and their predicted magnetic-field dependence using more precise tunnelling rate spectroscopy measurements^19^ as shown in Supplementary Fig. 1. The colour scale is shared with **d**.

To support the identification of the different relaxation channels discussed above, we carefully probe the state spectrum of our QD by standard Read window calibration^25,29^. In Fig. 2b, we plot the occupation probability of the QD estimated from ~140 single-shot traces, varying the Read level position relative to the alignment between the GS and the Fermi energy in the leads. The Load (*eV*_load_ = 95 μeV) and ‘Empty’ (*eV*_empty_ = −95 μeV) levels are kept constant during this process (see the sketch in Fig. 2a). The finite probability of having a step in the sensor trace reduces the average dot occupation at the beginning of the Read phase as seen from Fig. 2b (light-blue region). We can clearly distinguish three different regimes here: (I) low density of steps between 35 μeV and 65 μeV as we read out only the higher-energy ES |K^−^↓〉, (III) high density of steps between 0 and 35 μeV as we read out both of the ESs and (II) a very slow decay of the density of steps over time, indicating resonant back-and-forth jumps of the hole between the lower-energy ES |K^+^↓〉 and the lead as their energies align. In Fig. 2d we plot the beginning of the Read phase (within a time interval indicated by white arrows in Fig. 2b) at different out-of-plane magnetic fields. We clearly observe the predicted^7^ crossing between two ESs around *B*_⊥_ ≈ 80 mT. At zero magnetic field, the GS |K^−^↑〉 and the first ES |K^+^↓〉 form a Kramers pair and *B*_⊥_ splits it with *Δ*_1_ = (*g*_v_ + *g*_s_)*μ*_B_*B*_⊥_. Relaxation of the second ES |K^−^↓〉 into the GS is spin blocked and evolves in *B*_⊥_ as *Δ*_2_ = *g*_s_*μ*_B_*B*_⊥_ + *Δ*_SO_. The extracted *g*_v_ = 14.5 (we checked that *g*_s_ = 2 independently, Supplementary Fig. 1) and *Δ*_SO_ = 64 ± 4 μeV agree with the previously measured values^16–19^.

We can qualitatively compare the data with the results of a simple three-state Monte Carlo model of the single-shot readout^25^ (see Supplementary Section E for details) with ${T}_{1}^{\,({\rm{s}})}=0.5\,{\rm{s}}$ and ${T}_{1}^{\,({\rm{sv}})}=30\,{\rm{s}}$. Figure 2c shows the results of the simulations, which aim to qualitatively match the experimental step map in Fig. 2b. As expected, the simulation of the *B*_⊥_ dependence in Fig. 2e qualitatively agrees with the experiment in Fig. 2d.

## Identifying different relaxation channels

**Fig. 3:**
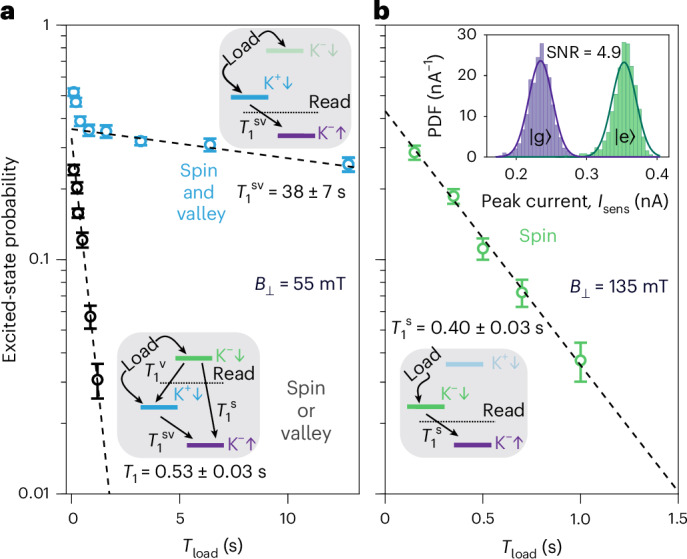
Distinguishing different relaxation channels. **a**, Exponential decays of the ES probability at *B*_⊥_ = 55 mT. The upper sketch shows the measurement of the relaxation time from both ESs in parallel (blue circles). Here, the Read level is placed below both of the the ESs and above the GS. The lower sketch depicts the relaxation measurements from only the higher-energy ES (black circles). Here, the Read level is placed between the first and the second ESs. Dashed lines show the exponential fits with given times *T*_1_. **b**, Exponential decay of the ES probability at *B*_⊥_ = 135 mT. The sketch depicts the standard spin readout protocol: the Load level is placed between the two ESs, while the Read level is placed between the GS and the lower-energy ES. The transition between these two states is spin blocked. Data points in **a**,**b** are presented as mean values ± the confidence interval of a single s.d. (Methods). Inset: probability density function (PDF) histograms of the maximum values of the sensor current in the Read phase extracted separately from the shots where the state at the beginning of the Read phase is identified as the ES (green) and the GS (purple). The solid lines are Gaussian fits obtained separately for each histogram. SNR, signal-to-noise ratio.

We start the discussion on how we access all three types of blockade in Fig. 1e with perpendicular magnetic fields beyond the crossing *B*_⊥_ > 80 mT. Here the relaxation between the first ES |K^−^↓〉 and the GS |K^−^↑〉 is spin blocked as depicted in Fig. 3b. During the Read phase, the *E*_F_ of the lead (represented by the dotted lines in Fig. 3) is placed between the GS and first ES to facilitate spin-to-charge conversion. The exponential decay of the spin-down probability in Fig. 3b yields a long ${T}_{1}^{\,({\rm{s}})}=0.40\pm 0.03\,{\rm{s}}$ (refs. ^3,4^).

At lower magnetic fields *B*_⊥_ < 80 mT, the ESs swap their order, and the relaxation between the now lower-energy ES |K^+^↓〉 and the GS |K^−^↑〉 becomes spin and valley blocked. Due to the presence of occasional charge jumps, we find it easier to load a hole into both ESs rather than accurately placing the Load level between the ESs. The lower sketch in Fig. 3a illustrates the readout of the higher-energy ES |K^−^↓〉, which requires a spin or valley flip to relax to the GS or the lower-energy ES, respectively. The resulting relaxation rate obtained from the black data points is, therefore, the average of two parallel relaxation channels ${T}_{1}={(1/{T}_{1}^{\,({\rm{s}})}+1/{T}_{1}^{\,({\rm{v}})})}^{-1}=0.53\pm 0.03\,{\rm{s}}$, with a value close to the spin relaxation times and probably dominated by it^3^. We want to highlight a key feature in the relaxation measurement of the higher-energy ES |K^−^↓〉: when loading times exceed *T*_load_ ≈ 2 s, all carriers loaded to the higher-energy ES relax, leaving it unoccupied.

Alternatively, we can measure relaxation from both the ESs by moving the Read level below the lower-energy ES, as shown in the upper sketch in Fig. 3a. Here, two distinct regimes in the exponential decay of the ES probability (blue data points) are evident: at short loading times the relaxation rate is fast and attributed to the relaxation from the highest ES, while around *T*_load_ > 1 s the second regime of rather long relaxation becomes apparent. As we have illustrated that the higher-energy ES is unoccupied at these loading times, we attribute the long ${T}_{1}^{\,({\rm{sv}})}=38\pm 7\,{\rm{s}}$ to the simultaneous spin–valley flip between the lower ES and the GS. The two-orders-of-magnitude difference in relaxation rates for different read levels is striking. We validate our observations and rule out potential thermally activated and flicker noise origin of steps in Methods.

## Conclusions

The notably long times *T*_1_, together with the demonstrated high-fidelity readout, present exciting opportunities for studying spin and valley physics at low and zero magnetic fields, where it holds particular significance as the energy splittings align with the capabilities of standard microwave equipment operating around 5–12 GHz. Performing such measurements in a device based on purified carbon-12 graphene would allow for a platform free from hyperfine-induced decoherence. Additionally, the much weaker spin-mixing component of a spin–orbit coupling in BLG (~5 neV)^3,30^, compared with other established semiconducting platforms (60 neV for GaAs^31^, 200 neV for Ge^32^ and 110 neV for silicon metal–oxide–semiconductor^33^), suggests that it may not limit spin coherence times in BLG due to charge noise, one of the key factors constraining the performance in conventional platforms^1^. Furthermore, replacing the lossy amorphous aluminium oxide between gate layers with dangling-bond-free hBN could potentially reduce charge noise in BLG QD devices^34^.

An open question remains regarding how to coherently manipulate the valley degree of freedom, since the valley magnetic moment is only coupled to the out-of-plane magnetic field. This problem can be addressed by utilizing spin–valley exchange interaction between the (2, 0) and (1, 1) orbital configurations in a double-dot geometry. The resulting Kramers singlet–triplet qubit can be operated similarly to its spin analogue^35,36^, leveraging the gate tunability of the valley *g* factor, as shown in Supplementary Fig. 4. Additionally, the readout of the Kramers singlet–triplet qubit can be achieved via Pauli spin–valley blockade^37^. Another approach to manipulating Kramers states relies on a presence of a finite valley-mixing term $\propto {\tau }_x{\Delta }_{{{\rm{K}}}^{+}{{\rm{K}}}^{-}}$ in the original QD Hamiltonian due to the breaking of translational symmetry^38^. As a result, spin–valley rotation can be driven by exploiting conventional electron spin resonance protocols (Supplementary Fig. 3) using antennas, micromagnets^1,2^ or the highly anisotropic effective *g* factor in a curved BLG QD^38^, similar to carbon nanotubes^20,39^. Independent of the magnitude of valley mixing, two-qubit gates between spin–valley qubits can be facilitated through exchange interactions, providing a robust roadmap for quantum information processing.

## Methods

### Device fabrication

All the presented data were measured on a single device fabricated on a van der Waals heterostructure stacked using standard dry transfer techniques. The stack consists of a 35-nm-thick top hBN layer, the Bernal BLG sheet, a 28-nm-thick bottom hBN and a graphite back gate layer. The ohmic contacts to the BLG are one-dimensional edge contacts. To form QDs, we utilize the two overlapping layers of Cr/Au (3 nm/20 nm) metallic gates shown in Fig. 1a. The upper gate layer consists of finger gates, 20 nm wide and 60 nm apart. These gates are deposited on top of a 26-nm-thick insulating aluminium oxide layer. The widths of the channels defined by the split gates are 40 nm for the left channel and 75 nm for the right channel. We tune the dot into the few-hole regime as shown in Fig. 1b. We attribute the set of parallel discrete steps to the boundaries of the regions of the charge stability diagram with a constant *N*_h_ = 0, 1, 2, … as indicated in the figure. We identify the first hole transition around *V*_P1_ ≈ 13 V (marked by the red circle), as no charge transition line appears at higher plunger gate voltages. Additional lines with markedly different couplings to MS and P1 gates correspond to unintended dots formed due to nearby charge inhomogeneities.

By applying a large negative voltage *V*_BG_ = −7.4 V to the global back gate we induce a large displacement field (*D* = −0.9 V nm^−1^), which opens a band gap in BLG of the order of 100 meV. This field allows us to notably decouple the dot from the reservoirs, achieving tunnelling rates to the leads of just tens of hertz.

### Measurement set-up

The sample is mounted on the mixing chamber of a Bluefors LD400 dilution refrigerator, which has a base temperature of 9 mK and an independently extracted electron temperature of around 30 mK. All the measurement and control electronics are located at room temperature and are connected to the device via 24 DC lines. Each line is low-pass filtered using *RC* filters mounted on the mixing chamber plate, with a time constant of approximately 10 μs. For DC biasing of gates and ohmic contacts, we use in-house-built low-noise voltage sources with a cutoff frequency of 7 Hz, except for the pulsing gate P1 line, which is left unfiltered and has a cutoff frequency of 1,200 Hz. The DC plunger gate voltage *V*_P1_ is combined with the pulsing voltage *V*_pulse_ using a 2 MΩ/2.7 kΩ divider at room temperature. The sensor current is amplified using an in-house-built current-to-voltage converter with a 10 MΩ feedback resistor, followed by a ×100 analogue voltage amplifier and an analogue low-pass filter with a cutoff frequency of around 10 kHz. Sensor time traces are recorded using an NI-6251 data acquisition card with a sampling frequency of 20 kHz.

### Elzerman sequence

We start the three-level single-shot readout sequence with the empty dot subjected to the Load phase (see Fig. 1c): the pulse level shifts all three states below the *E*_F_ of the leads. A hole from the lead can tunnel into any of the three states with almost equal probabilities^19^ of 1/3. During the Load phase of duration *T*_load_, the sensor current drops abruptly, indicating that the dot has been filled, as shown in the typical time traces in Fig. 1d. In the following Read phase (see Fig. 1c), the second pulse level puts the ES of interest above *E*_F_ in the lead while the GS stays below. If a hole remains in the ES after loading and during the Read phase without relaxing, then, at some random time governed by the tunnelling-out rate, it will tunnel to the leads and thereafter the GS of the dot will be occupied again through a tunnelling-in process. This in-and-out tunnelling manifests itself as a step in the sensor current shown in Fig. 1d (green trace). In contrast, no step is observed when the hole is initially loaded into the GS, or when it relaxes before it can tunnel out during the Read phase (blue trace). Once a hole arrives in the GS, it blocks any further transitions since the number of resonant unoccupied lead states is exponentially suppressed by the low *T*_e_ ≈ 30 mK (ref. ^19^). The final pulse level empties the dot by moving all states above *E*_F_ of the leads (see Fig. 1c), and after that the sequence starts again.

### Postselection of sensor traces

**Extended Data Fig. 1:**
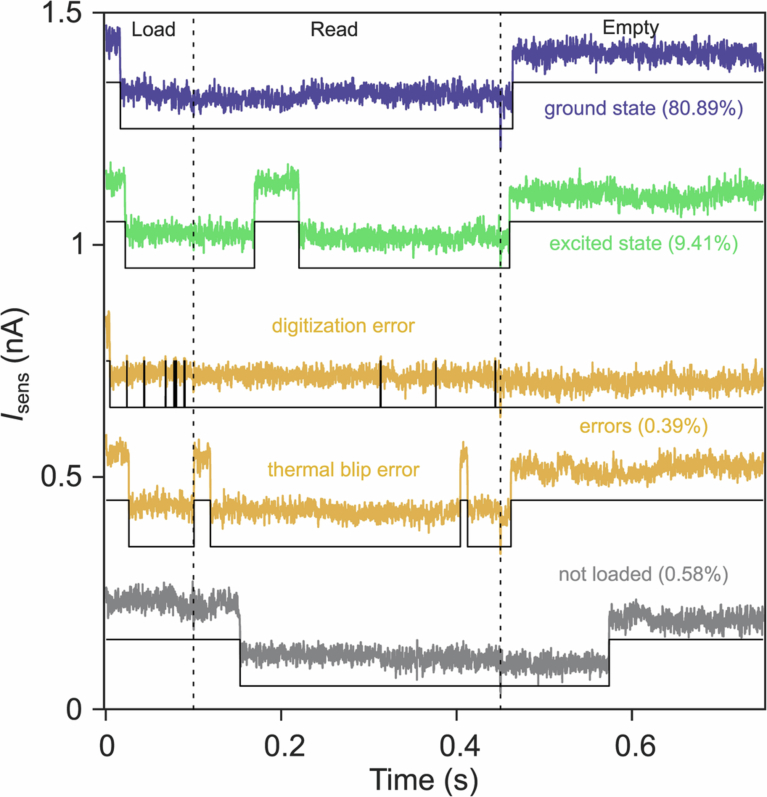
Postselection of sensor traces. Typical time traces of the sensor current during the three-level pulse. Traces are shifted vertically with an equal offset of 0.3 nA. Black lines show the digitized versions of the traces. By analyzing the digitized signals, we group all traces into four groups: excited states (green), ground states (purple), errors (yellow), or not loaded states (gray). The percentage breakdown for each group is specified.

We digitize current sensor traces using the two-threshold procedure described in ref. ^19^. We divide the entire set of *N* digitized current traces into four groups, *N* = (*N*_e_, *N*_g_, *N*_nl_, *N*_er_). Here, *N*_nl_ is the number of traces where a hole was not loaded during the Load phase. *N*_g_ is the number of traces with zero steps in the Read phase, which we label as a hole being in the GS. *N*_e_ is the number of traces with a single step during the Read phase, which we define as a hole being in the ES. *N*_er_ is for the rest of the traces, which we label as errors, as they exhibit multiple steps in the Read phase. Extended Data Fig. 1 shows a typical sensor current trace for each group along with its digitized version. The typical proportions are as follows: 80.89% for the GS, 9.41% for the ES, 0.39% for combined errors and 0.58% for traces that are not loaded. The two most common types of error causing multiple-step traces are digitization errors and random thermal/charge noise steps. The first is due to the fact that the sensor trace does not exhibit enough points in both charge states to reliably determine the threshold for digitization. Thermal steps are exponentially suppressed by low electron temperature, while major charge jumps occur on a very long timescale of minutes and hours. We simply discard all error traces from the dataset, assuming that the errors are uncorrelated with whether the ES or GS is occupied in the beginning of the Read phase. Note that by discarding clearly ES counts, although marked as thermal step errors in Extended Data Fig. 1, we underestimate the ES probability.

### Calculation of ES probability

**Extended Data Fig. 2:**
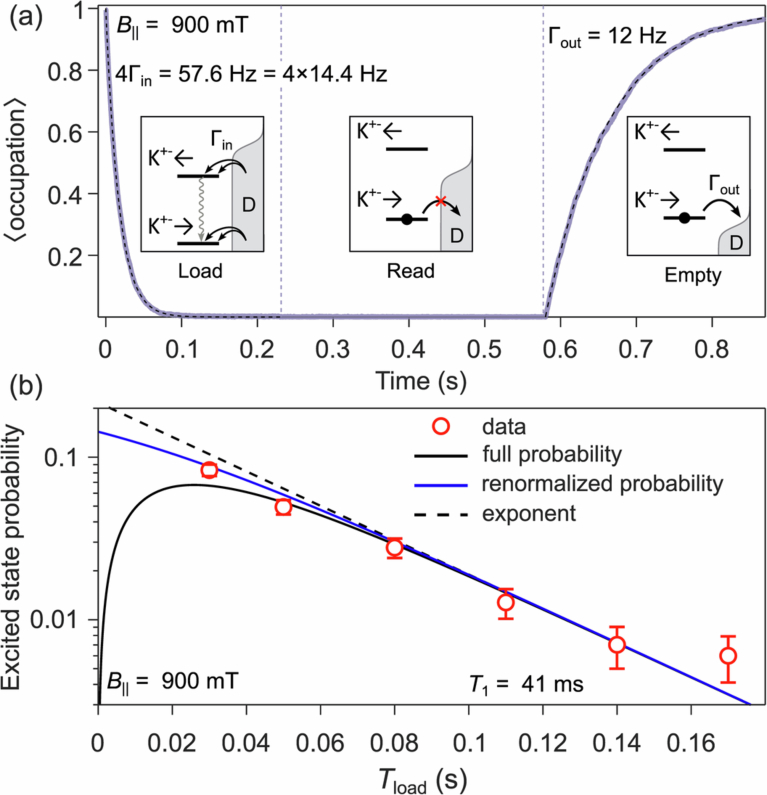
Calculation of excited state probability. (**a**) Averaged over approximately 2000 shots, the occupation of the QD is shown as a function of time for *B*_∥_ = 900 mT and a loading time *T*_load_ = 220 ms, notably longer than *T*_1_ = 41 ms. Dashed lines represent exponential fits with outlined tunneling rates *Γ*_in\out_. Energy diagrams sketch the standard three-level spin readout protocol. (**b**) The excited state probability is plotted against the loading time *T*_load_. The dashed line shows an exponential fit with *T*_1_ = 41 ms. The black line represents the full analytical probability, considering extracted tunneling rates and *T*_1_ = 41 ms. The blue solid line depicts the renormalized probability after excluding all non-loaded shots.

We perform relaxation time measurements in a regime with low *Γ*_out_ ≈ *Γ*_in_ ≈ 15 Hz, which are comparable to the measured *T*_1_ at certain magnetic fields. In this regime, the occupation of the ES after the short loading times *T*_load_ ≈ *Γ*_in_^−1^ is limited not by the relaxation but by the tunnelling-in rate. Nevertheless, even for one of the shortest extracted *T*_1_ ≈ 40 ms, we demonstrate that the decay of the calculated renormalized ES probability *P*^e^ can be fitted by a simple exponent. We consider the state readout of two double-degenerate Kramers pairs at *B*_∥_ = 900 mT, as shown in Extended Data Fig. 2a. As established in previous measurements^9,26^, the total occupations of the ES, GS and non-loaded states after *T*_load_ are

1 $$\begin{array}{rcl}&&{n}^{{\rm{e}}}({T}_{{\rm{load}}})=\frac{{N}_{{\rm{e}}}}{{N}_{{\rm{e}}}+{N}_{{\rm{g}}}+{N}_{{\rm{nl}}}}=\frac{{\varGamma }_{{\rm{in}}}^{{\rm{e}}}}{{\varGamma }_{{\rm{in}}}^{{\rm{e}}}+{\varGamma }_{{\rm{in}}}^{{\rm{g}}}-{T}_{1}^{-1}}\,{\mathrm{e}}^{-{T}_{{\rm{load}}}{T}_{1}^{-1}}\left(1-{\mathrm{e}}^{-{T}_{{\rm{load}}}({\varGamma }_{{\rm{in}}}^{{\rm{e}}}+{\varGamma }_{{\rm{in}}}^{{\rm{g}}}-{T}_{1}^{-1})}\right)\\ &&{n}^{{\rm{g}}}({T}_{{\rm{load}}})=\frac{{N}_{{\rm{g}}}}{{N}_{{\rm{e}}}+{Nas}_{{\rm{g}}}+{N}_{{\rm{nl}}}}=\frac{\left({\varGamma }_{{\rm{in}}}^{{\rm{g}}}-{T}_{1}^{-1}\right)\left(1-{\mathrm{e}}^{-{T}_{{\rm{load}}}({\varGamma }_{{\rm{in}}}^{{\rm{e}}}+{\varGamma }_{{\rm{in}}}^{{\rm{g}}})}\right)+{\varGamma }_{{\rm{in}}}^{{\rm{e}}}\left(1-{\mathrm{e}}^{-{T}_{{\rm{load}}}{T}_{1}^{-1}}\right)}{{\varGamma }_{{\rm{in}}}^{{\rm{e}}}+{\varGamma }_{{\rm{in}}}^{{\rm{g}}}-{T}_{1}^{-1}}\\ &&{n}^{{\rm{nl}}}({T}_{{\rm{load}}})=1-{n}^{{\rm{e}}}-{n}^{{\rm{g}}}\end{array}$$

where ${\varGamma }_{{\rm{in}}}^{{\rm{e}}}$ and ${\varGamma }_{{\rm{in}}}^{{\rm{g}}}$ are tunnelling-in rates to the ES and the GS respectively. We extract the sum of tunnelling-in rates ${\varGamma }_{{\rm{in}}}^{{\rm{e}}}+{\varGamma }_{{\rm{in}}}^{{\rm{g}}}=4{\varGamma }_{{\rm{in}}}=57.6\,{\rm{Hz}}$ and the tunnelling-out rate ${\varGamma }_{{\rm{out}}}^{{\rm{e}}}={\varGamma }_{{\rm{out}}}^{{\rm{g}}}={\varGamma }_{{\rm{out}}}=12\,{\rm{Hz}}$ by fitting the exponential decay and rise of the average dot occupation during the Load and Empty phases respectively, as shown in Extended Data Fig. 2a. During the Read phase, we measure the ESs with efficiency^9,26^

2 $${n}_{{\rm{RO}}}=\frac{{\varGamma }_{{\rm{out}}}^{{\rm{e}}}}{{T}_{1}^{-1}+{\varGamma }_{{\rm{out}}}^{{\rm{e}}}}\left(1-{\mathrm{e}}^{-{T}_{{\rm{read}}}({T}_{1}^{-1}+{\varGamma }_{{\rm{out}}}^{{\rm{e}}})}\right)$$

where *T*_read_ is the time spent in the Read phase and ${\varGamma }_{{\rm{out}}}^{{\rm{e}}}$ the intrinsic tunnelling rate.

The resulting full and renormalized probabilities are simply products of the two efficiencies:

3 $$\begin{array}{ll}&{P}_{{\rm{full}}}^{\,{\rm{e}}}({T}_{{\rm{load}}})=\frac{{N}_{{\rm{e}}}}{{N}_{{\rm{e}}}+{N}_{{\rm{g}}}+{N}_{{\rm{nl}}}}{n}_{{\rm{RO}}}={n}^{{\rm{e}}}{n}_{{\rm{RO}}}\\ &{P}_{{\rm{renorm}}}^{\,{\rm{e}}}({T}_{{\rm{load}}})=\frac{{N}_{{\rm{e}}}}{{N}_{{\rm{e}}}+{N}_{{\rm{g}}}}{n}_{{\rm{RO}}}=\frac{{n}^{{\rm{e}}}}{{n}^{{\rm{e}}}+{n}^{{\rm{g}}}}{n}_{{\rm{RO}}}.\end{array}$$

In Extended Data Fig. 2b, we plot the measured *P*^e^ = *N*_e_/(*N*_e_ + *N*_g_) along with theoretical curves from equation (3), using ${\varGamma }_{{\rm{in}}}^{{\rm{e}}}=22.2\,{\rm{Hz}}$ and *T*_1_ = 41 ms. As expected, at short *T*_load_, the full and renormalized occupations markedly differ. However, as long as ${T}_{{\rm{load}}} > 60\,{\rm{ms}}\approx 3/({\varGamma }_{{\rm{in}}}^{{\rm{e}}}+{\varGamma }_{{\rm{in}}}^{{\rm{g}}})$ the two curves match well, and their downward trend can be successfully approximated by the simple exponential function ${P}_{\exp }^{\,{\rm{e}}}({T}_{{\rm{load}}}) \approx {\mathrm{e}}^{-{T}_{{\rm{load}}}/{T}_{1}}$ as shown with the dashed line. With increasing relaxation time, the renormalized probability becomes closer to the exponent. The best least-square weighted exponential fit of the experimental points yields *T*_1_ = 45 ± 3 ms, which is within 10% of the value given by the correct renormalized probability expression.

### Dark counts

**Extended Data Fig. 3:**
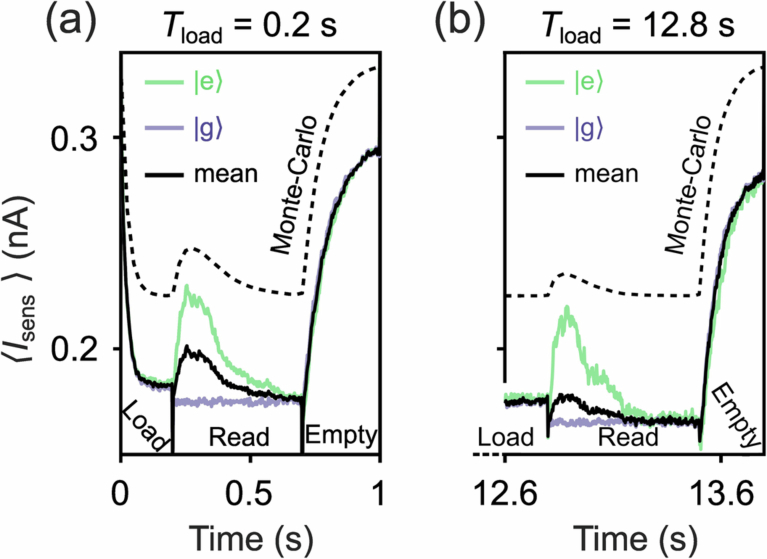
Dark counts. Average of single-shot sensor current traces identified as the ES (green), the GS (purple), and the average of both (black) are shown for two very distinct loading times *T*_load_ = 0.2 s (**a**) and 12.8 s (**b**) measured in *B*_⊥_ = 55 mT. Dashed lines show the mean of the traces generated via Monte Carlo simulations. The vertical offset for the simulated data is introduced for clarity.

In Extended Data Fig. 3, we rule out potential thermally activated and flicker noise origin (dark counts) of steps by comparing the step distribution at notably different *T*_load_ = 0.2 s and 12.8 s. The average of all the single-shot *I*_sens_ traces identified as the GS (purple) shows a flat behaviour during the Read phase. In contrast, the average of ESs (green) exhibits a bump that rises on a timescale of 1/*Γ*_out_ and decays within 1/*Γ*_in_ (ref. ^25^). The exponential decrease in step density to zero, governed by the tunnelling-out rate, allows us to eliminate the charge noise origin of the steps, as one would anticipate a constant distribution of random charge jumps over the Read phase. Another potential source of false-positive counts could be thermally activated steps. Since we only count single steps, the distribution might appear similar. However, with average tunnelling-in and out times of 1/*Γ*_in/out_ = 75 ms and a Read duration of *T*_read_ = 350 ms, we expect a Poissonian distribution *P*(*k*) = *λ*^*k*^ e^−*λ*^/*k*! for the probability of a certain number of tunnelling events *k*, with an average of *λ* = 4.7. The probability of having a single step (corresponding to two tunnelling events) is *P*(*k* = 2) ≈ 10%, while the probability of having no step is *P*(*k* = 0) ≈ 1%, meaning that the remaining 88% of shots should be discarded as errors with around 1% of all shots being not-loaded. In fact, for *T*_load_ = 0.2 s, we identify 45.9% of shots with a single step, 51.9% of shots with no steps, 0.9% as not-loaded shots and only 1.3% as multiple-step errors, effectively ruling out thermal activation as the origin of the steps.

### Readout performance

For quantum information applications, it is crucial to identify the factors limiting the readout performance. We analyse 2,000 single-shot traces to extract the histogram of the peak value of the sensor current during the Read phase, as illustrated in the inset of Fig. 3b. The well-separated Gaussian peaks representing the detection of the GS and the ES, with a signal-to-noise ratio of approximately 4.9, result in an electrical readout fidelity exceeding 99.9% (refs. ^25,27^). The negligible electrical readout error suggests that the readout performance is limited by the spin/valley-to-charge conversion^27^. We find that, despite the deliberate reduction of the tunnelling rates, the observed notably long spin–valley relaxation time still just meets all the minimum requirements^27^ for achieving a fault-tolerant 99% readout visibility threshold^40^, as follows. (1) A large energy splitting more than 13 times larger than electron temperature. In our experiment *Δ*_1_ ≈ 55 μeV ≳ 13*k*_B_*T* ≈ 52 μeV. (2) A tunnelling-out time 100 times faster than the relaxation time. In our experiment, ${T}_{1}^{({\rm{sv}})}=30\,{\rm{s}}\gtrsim 100/{\Gamma }_{{\rm{out}}}=8\,{\rm{s}}$. (3) A sampling rate for data acquisition 12 times larger than the reloading rate. In our experiment, *Γ*_s_ = 10 kHz > 12*Γ*_out_ = 150 Hz.

### Ruling out pure spin relaxation

Here we explicitly rule out the argument that the observed long spin–valley relaxation times could be interpreted as fully dominated by pure spin relaxation. Indeed, spin *T*_1_ times from a few seconds to almost a minute^10^ have also been observed in both silicon and GaAs QDs^41^. Moreover, previous studies do indeed report strong power-law dependences^41^, which could, at first sight, explain the marked drop in relaxation times as we transition from the pure spin to the spin–valley blockade regime while simultaneously shrinking the energy splitting.

**Extended Data Fig. 4:**
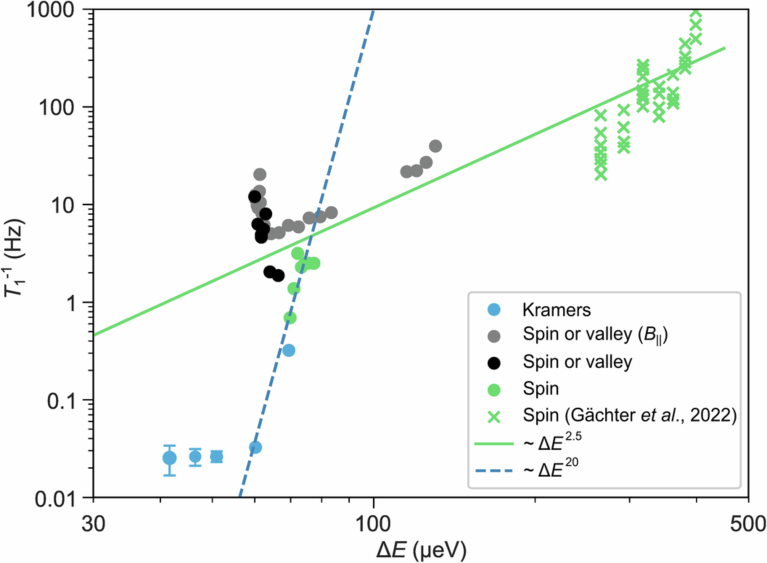
Ruling out pure spin relaxation. Measured inverse relaxation time ${{{{T}}}_{1}}^{-1}$ as a function of the energy splitting between relevant states. Circular markers of different colors correspond to different relaxation channels as outlined in the legend. Crosses correspond to the data points from Gächter et al.^4^, measured with the same Elzerman technique in a weakly coupled single BLG QD. Solid line shows the ~ *Δ*E^2.5^ trend in spin relaxation data. Dashed line shows ~ *Δ*E^20^, ruling out the assumption that Kramers qubit relaxation is dominated by the spin channel.

To eliminate this argument, in Extended Data Fig. 4 we plot the data from Fig. 1e as a function of the energy splitting between the first ES and the GS. Additionally, we include the spin relaxation rate *T*_1_^−1^ data at higher magnetic fields (1.5–3 T) from ref. ^4^, measured in an analogous weakly coupled (*Γ* ≈ 350 Hz) single-QD BLG device using the same Elzerman technique. The energy splitting dependence of *T*_1_ extracted from both experiments is best described by a power law, *T*_1_^−1^ ∝ Δ*E*^2.5^ (highlighted by the green solid line). The observed power is in line with ∝Δ*E*^3−7^, as seen in GaAs and silicon spin qubits, and originates from a combination of electron–phonon relaxation and various spin-mixing mechanisms such as hyperfine interaction, spin–orbit coupling and spin–valley mixing^41^. In the case of BLG, spin-mixing mechanisms are not well known, nor has any theoretical prediction for spin *T*_1_ dependence on the magnetic field been made while considering the two-dimensional nature of phonons. However, calculations for single-layer graphene show that the power law *T*_1_^−1^ ∝ Δ*E*^2–4^ should not differ much from the mentioned three-dimensional platforms. Taking this into account, if we assume that long spin–valley relaxation is solely dominated by spin relaxation, the trend correlating spin and Kramers points reveals a remarkable power of ∝Δ*E*^20^ as marked by the dashed blue line, although fitting a power law on such a restricted energy range should be approached with caution. Hence, a more plausible explanation for such a marked change in *T*_1_ would be the dual protection afforded by simultaneous spin–valley blocking when operating within the Kramers doublet. The continuous connection between the spin–valley (blue dots) and spin (green dots) relaxation data points can be attributed to the finite valley mixing term. Indeed, even small values of ${\Delta }_{{{\rm{K}}}^{+}{{\rm{K}}}^{-}} < 2\,{\rm{neV}} \approx 0.5\,{\rm{MHz}}$ (ref. ^3^) can provide effective mixing, considering that our shortest loading times are in the tens of milliseconds. Additionally, the spin or valley relaxation channel data points align with the pure spin trend, indicating that valley relaxation is significantly longer than spin relaxation, in agreement with previous observations^3^.

## Online content

Any methods, additional references, Nature Portfolio reporting summaries, source data, extended data, supplementary information, acknowledgements, peer review information; details of author contributions and competing interests; and statements of data and code availability are available at 10.1038/s41565-025-01858-8.

## Supplementary information

Supplementary Information Supplementary Figs. 1–5 and Discussion Sections A–F.

## Data Availability

The data that support the findings of this study will be made available online through the ETH Research Collection^42^ at http://hdl.handle.net/20.500.11850/711208. This includes raw data, analysis scripts and plotting scripts for figures from the main text, Extended Data and Supplementary Information.

## References

[1] Burkard, G., Ladd, T. D., Pan, A., Nichol, J. M. & Petta, J. R. Semiconductor spin qubits. *Rev. Mod. Phys.* **95**, 025003 (2023).

[2] Chatterjee, A. et al. Semiconductor qubits in practice. *Nat. Rev. Phys.* **3**, 157–177 (2021).

[3] Garreis, R. et al. Long-lived valley states in bilayer graphene quantum dots. *Nat. Phys.* **20**, 428–434 (2024).

[4] Gächter, L. M. et al. Single-shot spin readout in graphene quantum dots. *PRX Quantum* **3**, 020343 (2022).

[5] Banszerus, L. et al. Particle–hole symmetry protects spin–valley blockade in graphene quantum dots. *Nature* **618**, 51–56 (2023). 37138084 10.1038/s41586-023-05953-5

[6] McCann, E. & Koshino, M. The electronic properties of bilayer graphene. *Rep. Prog. Phys.* **76**, 056503 (2013). 23604050 10.1088/0034-4885/76/5/056503

[7] Knothe, A., Glazman, L. I. & Fal’ko, V. I. Tunneling theory for a bilayer graphene quantum dot’s single- and two-electron states. *New J. Phys.* **24**, 043003 (2022).

[8] Kormányos, A., Zólyomi, V., Drummond, N. D. & Burkard, G. Spin–orbit coupling, quantum dots, and qubits in monolayer transition metal dichalcogenides. *Phys. Rev. X* **4**, 011034 (2014).

[9] Borjans, F., Zajac, D. M., Hazard, T. M. & Petta, J. R. Single-spin relaxation in a synthetic spin–orbit field. *Phys. Rev. Appl.* **11**, 044063 (2019).

[10] Camenzind, L. C. et al. Hyperfine-phonon spin relaxation in a single-electron GaAs quantum dot. *Nat. Commun.* **9**, 3454 (2018). 30150721 10.1038/s41467-018-05879-x PMC6110844

[11] Wang, L. et al. One-dimensional electrical contact to a two-dimensional material. *Science* **342**, 614 (2013). 24179223 10.1126/science.1244358

[12] Krishnan, R. et al. Spin–valley locking for in-gap quantum dots in a MoS_2_ transistor. *Nano Letters* **23**, 6171 (2023). 37363814 10.1021/acs.nanolett.3c01779

[13] Knothe, A. & Fal’ko, V. Influence of minivalleys and Berry curvature on electrostatically induced quantum wires in gapped bilayer graphene. *Phys. Rev. B* **98**, 155435 (2018).

[14] Tong, C. et al. Tunable valley splitting and bipolar operation in graphene quantum dots. *Nano Letters* **21**, 1068 (2021). 33449702 10.1021/acs.nanolett.0c04343

[15] Kane, C. L. & Mele, E. J. Quantum spin Hall effect in graphene. *Phys. Rev. Lett.* **95**, 226801 (2005). 16384250 10.1103/PhysRevLett.95.226801

[16] Kurzmann, A. et al. Kondo effect and spin–orbit coupling in graphene quantum dots. *Nat. Commun.* **12**, 6004 (2021). 34650056 10.1038/s41467-021-26149-3 PMC8516925

[17] Banszerus, L. et al. Spin–valley coupling in single-electron bilayer graphene quantum dots. *Nat. Commun.* **12**, 5250 (2021). 34475394 10.1038/s41467-021-25498-3 PMC8413270

[18] Banszerus, L. et al. Spin relaxation in a single-electron graphene quantum dot. *Nat. Commun.* **13**, 3637 (2022). 35752620 10.1038/s41467-022-31231-5 PMC9233672

[19] Duprez, H. et al. Spin-valley locked excited states spectroscopy in a one-particle bilayer graphene quantum dot. *Nat. Commun.* **15**, 9717 (2024). 39521761 10.1038/s41467-024-54121-4 PMC11550441

[20] Laird, E. A., Pei, F. & Kouwenhoven, L. P. A valley–spin qubit in a carbon nanotube. *Nat. Nanotechnol.* **8**, 565–568 (2013). 23892984 10.1038/nnano.2013.140

[21] Churchill, H. O. H. et al. Relaxation and dephasing in a two-electron ^13^C nanotube double quantum dot. *Phys. Rev. Lett.* **102**, 166802 (2009). 19518737 10.1103/PhysRevLett.102.166802

[22] Kuemmeth, F., Ilani, S., Ralph, D. C. & McEuen, P. L. Coupling of spin and orbital motion of electrons in carbon nanotubes. *Nature* **452**, 448–452 (2008). 18368113 10.1038/nature06822

[23] Icking, E. et al. Transport spectroscopy of ultraclean tunable band gaps in bilayer graphene. *Adv. Electron. Mater.* **8**, 2200510 (2022).

[24] Elzerman, J. M. et al. Single-shot read-out of an individual electron spin in a quantum dot. *Nature* **430**, 431–435 (2004). 15269762 10.1038/nature02693

[25] Morello, A. et al. Single-shot readout of an electron spin in silicon. *Nature* **467**, 687–691 (2010). 20877281 10.1038/nature09392

[26] Simmons, C. B. et al. Tunable spin loading and *T*_1_ of a silicon spin qubit measured by single-shot readout. *Phys. Rev. Lett.* **106**, 156804 (2011). 21568595 10.1103/PhysRevLett.106.156804

[27] Keith, D. et al. Benchmarking high fidelity single-shot readout of semiconductor qubits. *New J. Phys.* **21**, 063011 (2019).

[28] Amasha, S. et al. Electrical control of spin relaxation in a quantum dot. *Phys. Rev. Lett.* **100**, 046803 (2008). 18352316 10.1103/PhysRevLett.100.046803

[29] Yang, C. H. et al. Spin–valley lifetimes in a silicon quantum dot with tunable valley splitting. *Nat. Commun.* **4**, 2069 (2013). 23804134 10.1038/ncomms3069

[30] Konschuh, S., Gmitra, M., Kochan, D. & Fabian, J. Theory of spin–orbit coupling in bilayer graphene. *Phys. Rev. B* **85**, 115423 (2012).

[31] Petta, J. R., Lu, H. & Gossard, A. C. A coherent beam splitter for electronic spin states. *Science* **327**, 669 (2010). 20133567 10.1126/science.1183628

[32] Jirovec, D. et al. Dynamics of hole singlet–triplet qubits with large *g*-factor differences. *Phys. Rev. Lett.* **128**, 126803 (2022). 35394319 10.1103/PhysRevLett.128.126803

[33] Tanttu, T. et al. Controlling spin–orbit interactions in silicon quantum dots using magnetic field direction. *Phys. Rev. X* **9**, 021028 (2019).

[34] Wang, J. I.-J. et al. Hexagonal boron nitride as a low-loss dielectric for superconducting quantum circuits and qubits. *Nat. Mater.* **21**, 398–403 (2022). 35087240 10.1038/s41563-021-01187-w

[35] Petta, J. R. et al. Coherent manipulation of coupled electron spins in semiconductor quantum dots. *Science* **309**, 2180 (2005). 16141370 10.1126/science.1116955

[36] Jirovec, D. et al. A singlet–triplet hole spin qubit in planar Ge. *Nat. Mater.* **20**, 1106–1112 (2021). 34083775 10.1038/s41563-021-01022-2

[37] Tong, C. et al. Pauli blockade of tunable two-electron spin and valley states in graphene quantum dots. *Phys. Rev. Lett.* **128**, 067702 (2022). 35213193 10.1103/PhysRevLett.128.067702

[38] Flensberg, K. & Marcus, C. M. Bends in nanotubes allow electric spin control and coupling. *Phys. Rev. B* **81**, 195418 (2010).

[39] Pei, F., Laird, E. A., Steele, G. A. & Kouwenhoven, L. P. Valley–spin blockade and spin resonance in carbon nanotubes. *Nat. Nanotechnol.* **7**, 630–634 (2012). 23001302 10.1038/nnano.2012.160

[40] Mills, A. et al. High-fidelity state preparation, quantum control, and readout of an isotopically enriched silicon spin qubit. *Phys. Rev. Appl.* **18**, 064028 (2022).

[41] Stano, P. & Loss, D. Review of performance metrics of spin qubits in gated semiconducting nanostructures. *Nat. Rev. Phys.* **4**, 672–688 (2022).

[42] Denisov, A. et al. Spin–valley protected Kramers pair in bilayer graphene. *ETH-Bibliothek* https://doi.org/10.3929/ethz-b-000711208 (2025). 10.1038/s41565-025-01858-8 PMC12015173 39930102

