# Supplementary material for: Spin–valley protected Kramers pair in bilayer graphene
Source: Nat Nanotechnol. 2025 Feb 10;20(4):494–9. doi: 10.1038/s41565-025-01858-8 (PMC12015173; doi:10.1038/s41565-025-01858-8)
Supplement: Supplementary file 1 — Supplementary Figs. 1–5 and Discussion Sections A–F. [file 41565_2025_1858_MOESM1_ESM.pdf]

nature nanotechnology

Supplementary information https://doi.org/10.1038/s41565-025-01858-8

Spin–valley protected Kramers pair in bilayer graphene

In the format provided by the authors and unedited

Supplementary Information

CONTENTS

A. Tunneling rate spectroscopy 2

B. Spectroscopy around relaxation hotspots 3

C. Coherent manipulation of the Kramers states 3
Kramers singlet-triplet qubit 3
Loss-DiVincenzo Kramers qubit via finite intervalley mixing 4
Valley qubit via finite intervalley mixing 4

D. Gate-tunable valley magnetic moment 6

E. Monte Carlo simulations 7
Single-shot readout in a three-state quantum dot 7
Probability of tunneling 7

F. Charge stability diagram 9

References 9

A. TUNNELING RATE SPECTROSCOPY

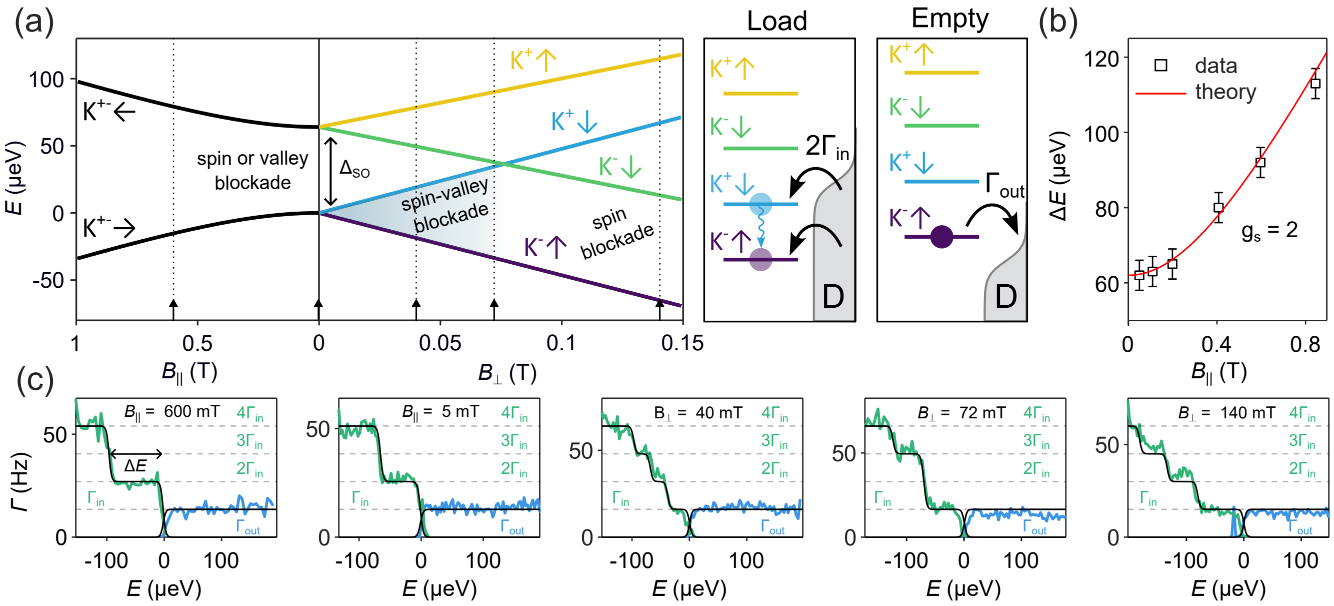

FIG. 1. **Tunneling rate spectroscopy.** (a) Energy spectrum of a single carrier in the BLG QD plotted as a function of in-plane and out-of-plane magnetic fields. Two-level pulse sequence for determining the in- and out-tunneling rates. The tunneling-in rate is proportional to the number of available energy states, while the tunneling-out rate is independent of the carrier's state. (b) Energy splitting between Kramers pairs ΔE is plotted as a function of in-plane magnetic field $B_{||}$. Solid red line is a theoretical curve with $g_s = 2$ and $\Delta_{SO} = 62$ μeV. Data points are presented as mean values $\pm$ the thermal broadening $3.5 k_B T_e/2 \approx$ 4 μeV of the Fermi-Dirac function. (c) Measured at different magnetic fields, tunneling-in (green) and -out (blue) rates are plotted as a function of the energy shift E relative to the GS. Black solid lines show theoretical curves with the sum of Fermi-Dirac distribution functions.

Tunneling rate spectroscopy technique assumes that all four states share the same orbital wave function. Consequently, the change in tunneling-in rates can be viewed as consecutive step functions, where the number of steps corresponds to the number of available states. To effectively measure the tunneling rates, we apply slow $(1-3$ Hz) periodic two-level square pulses which aim to 'Load' and 'Empty' the dot in each cycle as sketched in Fig. 1a. By measuring the waiting time t between the pulse and the actual tunneling event, we can extract both tunneling rates by fitting the exponential distribution $\sim \exp\{(-\Gamma_{\mathrm{in(out)}} t)\}$. Additional details can be found in [1], where similar measurements were conducted on the same device. Fig. 1a shows the spectrum of the single hole in BLG QD as a function of in- and out-of-plane magnetic fields [2]. Close to zero magnetic field, two doubly-degenerate Kramers pairs manifest themselves as two plateaus in measured tunneling-in rate at approximately $\Gamma_{\mathrm{in}} = 2\Gamma_{\mathrm{out}}$ and $\Gamma_{\mathrm{in}} = 4\Gamma_{\mathrm{out}}$ as shown in Fig. 1c for $B_{||} = 5\,\mathrm{mT}$. As expected, the tunneling-out rate is constant $\Gamma_{\mathrm{out}} \approx 13\,\mathrm{Hz}$ far away from the transition. The width of the $2\Gamma_{\mathrm{in}}$ step at zero magnetic field corresponds to the spin–orbit gap Δ_{SO}. As we increase the in-plane field to $B_{||} = 600\,\mathrm{mT}$, the degeneracy of the doublets stays the same, while the energy gap is increasing according to the in-plane polarization of the spin $\Delta = \sqrt{\Delta_{\mathrm{SO}}^2 + (g_s \mu B_{||})^2}$. We fit the data with a sum of Fermi-Dirac distribution functions [1] with electron temperature $T_e = 25\,\mathrm{mK}$ and $\Delta_{\mathrm{SO}} = 62\,\mu\mathrm{eV}$ and $g_v = 14.5$. In contrast, the perpendicular magnetic field disrupts the degeneracy of the doublets. At $B_\perp = 40\,\mathrm{mT}$, we notice four nearly evenly spaced energy steps, each with $\Gamma_{\mathrm{in}} = \Gamma_{\mathrm{out}}$. As we increase the field, at $B_\perp = 80\,\mathrm{mT}$, two ESs intersect, leading to the merging of the second and third plateaus into a single one with $\Gamma_{\mathrm{in}} = 3\Gamma_{\mathrm{out}}$ as evident from the data. However, beyond the crossing point, at $B_\perp = 140\,\mathrm{mT}$, the second plateau reappears with $\Gamma_{\mathrm{in}} = 2\Gamma_{\mathrm{out}}$. Note that we were only able to tune the dot to this textbook spectrum when the tunneling rates were as low as $10-20$ Hz.

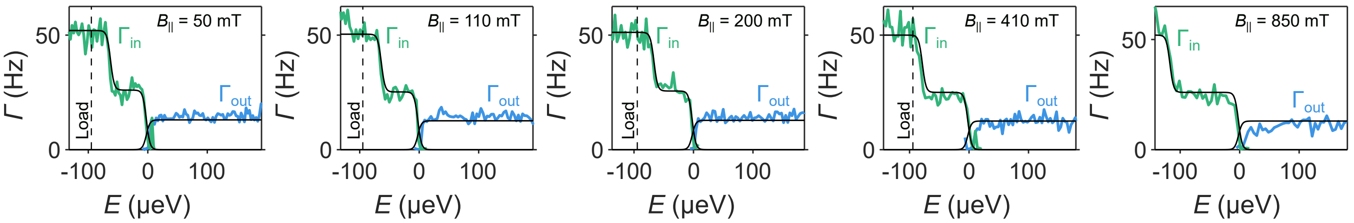

FIG. 2. **Tunneling rate spectroscopy around relaxation hotspot.** Measured at different in-plane magnetic fields $B_{||}$, tunneling-in (green) and -out (blue) rates are plotted as a function of the energy shift E relative to the GS. Black solid lines show analytical curves with the sum of Fermi-Dirac distribution functions, $g_s = 2$ and $\Delta_{\rm SO} = 60$ μeV. The loading levels are marked with the dashed lines.

B. SPECTROSCOPY AROUND RELAXATION HOTSPOTS

We noticed a sharp but rather shallow (2-3 times drop) hotspot in the measured T_1 around $B_{||} \approx 110\,$mT. In Fig. 2, we show the results of the tunneling rate spectroscopy around this point. As expected, all tunneling-in rate curves exhibit a similar two-step behavior as a function of the energy shift E in respect to the Fermi level in the leads. The width of the first step is defined by the splitting between two Kramers pairs and matches well with the theoretical predictions $\Delta E = \sqrt{\Delta_{\rm SO}^2 + (g_{\rm s}\mu_{\rm B}B_{||})^2}$, where $\Delta_{\rm SO} = 60\,\mu$eV and $g_{\rm s} = 2$. The only thing we can point out at $B_{||} = 110\,$mT (right on the hotspot) is a sign of a higher excited state emerging as an additional step in $\Gamma_{\rm in}$ at $E < -100\,\mu$eV. However, this state is lying higher than the loading level, outlined by the dashed line, and should not contribute to the measurements.

C. COHERENT MANIPULATION OF THE KRAMERS STATES

Kramers singlet-triplet qubit

For a double quantum dot with a single carrier in each dot (1,1), the ground state is any combination of the following 4 states: $|K^+\downarrow, K^+\downarrow\rangle$, $|K^-\uparrow, K^-\uparrow\rangle$, $|K^+\downarrow, K^-\uparrow\rangle$, $|K^-\uparrow, K^+\downarrow\rangle$.

In the singlet-triplet basis:

$$
\begin{aligned}
|A\rangle(1,1) &= \frac{1}{\sqrt{2}}\big(|K^+\downarrow, K^-\uparrow\rangle - |K^-\uparrow, K^+\downarrow\rangle\big)\\
|B_0\rangle(1,1) &= \frac{1}{\sqrt{2}}\big(|K^+\downarrow, K^-\uparrow\rangle + |K^-\uparrow, K^+\downarrow\rangle\big)\\
|B_+\rangle(1,1) &= |K^+\downarrow, K^+\downarrow\rangle\\
|B_-\rangle(1,1) &= |K^-\uparrow, K^-\uparrow\rangle
\end{aligned}
$$

In the case of two-particles in a single dot, the ground state at B=0 has been shown to be a triple-degenerate valley-singlet, spin-triplet state [3]:

$$
\begin{aligned}
|S^{\rm v}T_0^{\rm s}\rangle(2,0) &= \frac{1}{2}\big(|K^+\uparrow, K^-\downarrow\rangle - |K^-\downarrow, K^+\uparrow\rangle + |K^+\downarrow, K^-\uparrow\rangle - |K^-\uparrow, K^+\downarrow\rangle\big)\\
|S^{\rm v}T_+^{\rm s}\rangle(2,0) &= \frac{1}{\sqrt{2}}\big(|K^+\uparrow, K^-\uparrow\rangle - |K^-\uparrow, K^+\uparrow\rangle\big)\\
|S^{\rm v}T_-^{\rm s}\rangle(2,0) &= \frac{1}{\sqrt{2}}\big(|K^+\downarrow, K^-\downarrow\rangle - |K^-\downarrow, K^+\downarrow\rangle\big)
\end{aligned}
$$

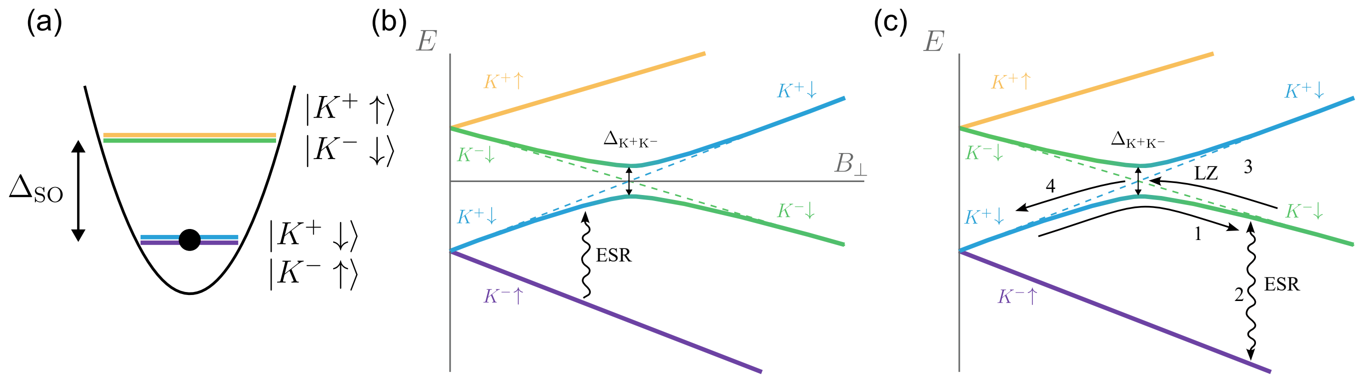

FIG. 3. **Kramers qubit manipulation based on valley-mixing.** (a) Single carrier QD in BLG. (b) Energy dispersion of a single carrier in BLG QD in the presence of a finite intervalley scattering term $\Delta_{K^+K^-}$. The Kramers qubit can be driven via Electric Spin Resonance (ESR).

As we see, only $|S^{\mathrm{v}}T^{\mathrm{s}}_0\rangle(2,0)$ and $|A\rangle(1,1)$ (highlighted in blue) can be coupled by interdot tunnelling in the assumption that the spin and valley quantum numbers are conserved during the tunnelling process. One can show that the resulting tunneling Hamiltonian, spanned by the Kramers singlet $|A\rangle$(1,1) and unpolarized triplet $|B_0\rangle(1,1)$ states is similar to its spin analog [4].

Loss-DiVincenzo Kramers qubit via finite intervalley mixing

In the presence of a finite valley scattering term, the operation of the Kramers qubit is relatively straightforward, as theoretically described in [5] as well as experimentally demonstrated on carbon nanotubes by Laird et al. [6]. Unlike CNTs, however, $\Delta_{K^+K^-} < 0.5$ MHz in BLG is significantly suppressed due to the low long- and short-range disorder which would otherwise cause large-momentum scattering. Following the theoretical work on CNTs, in a presence of the finite intervalley mixing, we can project our original single particle Hamiltonian:

$$H = -\frac{1}{2}\left(\tau_{\mathrm{z}}\Delta_{\mathrm{SO}}\sigma_{\mathrm{z}} + \tau_{\mathrm{x}}\Delta_{K^+K^-}\right) + g_{\mathrm{s}}\mu_{\mathrm{B}}\vec{\sigma}\cdot\vec{B} + \tau_{\mathrm{z}}g_{\mathrm{v}}\mu_{\mathrm{B}}B_\perp$$

onto the lowest two eigenstates (Kramers pair) yielding an effective spin-1/2 system:

$$H^* = \frac{1}{2}\mu_B\left(s_{\mathrm{z}}g_\perp B_\perp + s_{\mathrm{x}}g_{||}B_{||}\right), \quad \text{where } g_\perp = g_{\mathrm{s}} + 2g_{\mathrm{v}}\frac{\Delta_{\mathrm{SO}}}{\sqrt{\Delta^2_{\mathrm{SO}} + \Delta^2_{K^+K^-}}}, \quad g_{||} = 2g_{\mathrm{s}}\frac{\Delta_{K^+K^-}}{\sqrt{\Delta^2_{\mathrm{SO}} + \Delta^2_{K^+K^-}}}$$

Here, s_{z} and s_{z} are spin Pauli matrices, and $B_{||}$ represents the in-plane magnetic field parallel to the BLG plane. The above Hamiltonian allows for the use of conventional ESR to drive (see Fig. 3b) the qubits, either by employing a microwave antenna or micromagnet, or by utilizing the geometric bends of the QD, as demonstrated in CNTs [6]. Note that the desirable in-plane g-factor component $g_{||}$, is expected to vanish as $\Delta_{\mathrm{K^+K^-}} \to 0$. This originates from the mixing of the first $K^+\downarrow$ and second $K^-\downarrow$ excited states as we approach the anticrossing point, as shown in Fig. 3b. In BLG, $\Delta_{\mathrm{K^+K^-}} \ll \Delta_{\mathrm{SO}}$, so $g_{||} \ll g_{\mathrm{s}}$ and as a result, Rabi frequencies are expected to be significantly slower than those of spin Loss-DiVincenzo qubits [7].

Valley qubit via finite intervalley mixing

Another interesting possibility is to drive a pure valley qubit using a non-adiabatic pulsing across the Landau-Zener transition shown in Fig. 3c. Fast pulsing across the magnetic field axis can be achieved by leveraging the tunable valley g-factor. One possible protocols is illustrated in Fig. 3c:

1. Adiabatically drive the system across the LZ transition, to obtain a quantum state encoded solely in the spin, and perform Elzerman readout to initialize the spin in the ground state.

2. Apply a spin π-pulse using ESR drive to excite the carrier into $K^- \downarrow$ state.
3. Non-adiabatically pulse close to the anticrossing and wait for Larmor precession of the valley with rate equal to $\Delta_{\mathrm{K^+K^-}}$.
4. Non-adiabatically pulse far from the anticrossing and perform energy-selective readout between $K^- \downarrow$ and $K^+ \downarrow$ states.

D. GATE-TUNABLE VALLEY MAGNETIC MOMENT

The unique property of BLG is a gate-tunable valley g-factor, which allows electrical driving of the Kramers singlet-triplet states as well as adiabatic sweeps across the intervalley (anti)crossing. Here we experimentally demonstrate the tunability of g_v in the range from approximately 10 to 40 by simply varying the split-gate voltage. In Fig. 4 we measured the transition between 4 and 5 electrons in the QD, which behave similarly to the 0-to-1 transition, as 4 carriers form a full orbital shell. We did not observe this effect for the 0-to-1 transition within the range of split-gate voltages where the excited states of the dot are still resolvable. We attribute this to the fact that a quantum dot with 4 electrons is inherently larger than one starting with 0 electrons, and the size is what defines the valley magnetic moment [8].

In Fig. 4a, a Coulomb diamond is measured via direct current through the QD (high tunnelling rates). Finite-bias spectroscopy in Fig. 4b reveals the canonical single-particle energy spectrum as a function of the out-of-plane magnetic field, including two Kramers pairs. At fixed perpendicular magnetic field $B_\perp = 75$ mT, we track the energy splitting (red arrow in Fig. 4b) between $K^- \downarrow$ (green) and $K^+ \downarrow$ (blue) states as a function of split-gate voltage as shown in Fig. 4c. To directly measure the energy splitting, we sweep the bias voltage rather than the plunger gate, avoiding the need to separately measure the lever arm for each split-gate voltage. We can clearly observe the two excited states moving closer together and eventually crossing as the split-gate voltage is slightly increased (1.5 mV). This is directly related to the change of valley g-factor and moving of the crossing point between $K^- \downarrow$ (green) and $K^+ \downarrow$ (blue) states to higher magnetic fields in Fig. 4b (from 30 mT to 115 mT). The position of this crossing is defined only by the spin-orbit gap Δ_{SO} and valley g-factor g_v, while we ensure that the SO gap remains unchanged as a function of the split-gate voltage. This tunability enables nonadiabatic pulsing across the crossing point using electrical signals as well as ESR-like driving of the singlet-triplet qubit. Since the split-gate also influences the transition in plunger gate voltage, we virtually compensate for the plunger gate voltage to stay always around the the transition in Fig. 4c. This linear compensation is responsible for non-monotonic trend of excited state lines.

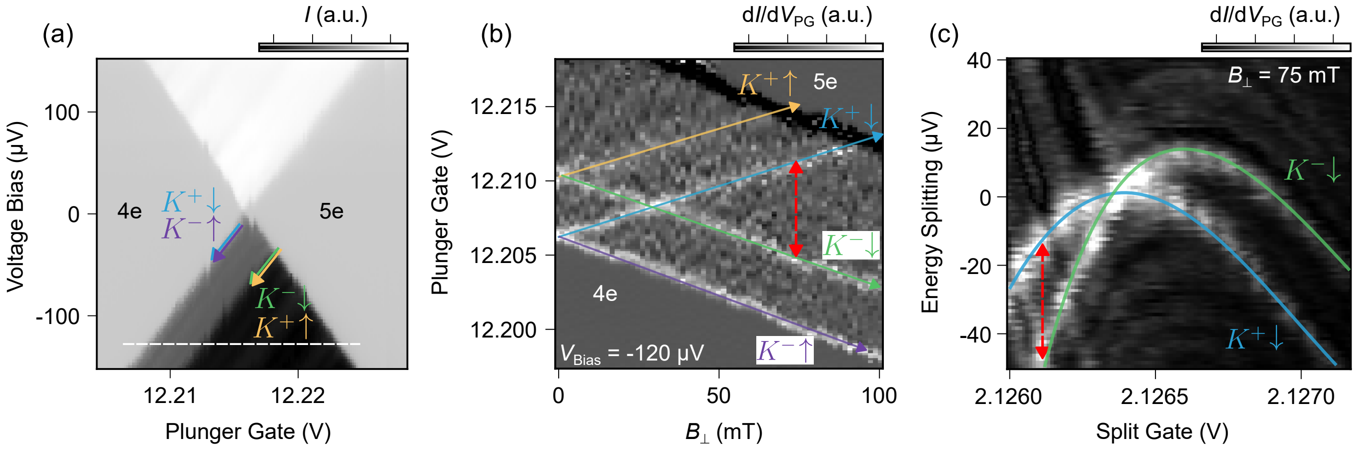

FIG. 4. **Tunable valley magnetic moment.** (a) The Coulomb diamond measured in the direct current through the quantum dot (QD) between the 4-to-5 electrons transition. The white dashed line indicates the bias voltage used for spectroscopy in a magnetic field. (b) Finite-bias spectroscopy as a function of the out-of-plane magnetic field shows the canonical spectrum. (c) The energy splitting between the first and second excited states is presented as a function of the split gate, measured at a fixed magnetic field of $B_\perp = 75$ mT. The reverse crossing of the states indicates the high tunability of the valley g-factor. Solid lines are provided as guides for the eye.

E. MONTE CARLO SIMULATIONS

Single-shot readout in a three-state quantum dot

In our simulation, we considered the quantum dot to be a 3-level system, with ground state, first excited state, and second excited state. The whole process of readout is divided into 4 parts - the loading stage, the waiting stage, the measurement stage, and the unloading stage.

In the loading stage, a charge carrier is loaded into the quantum dot. Because all the 3 states are well below the electrochemical potential of the leads, we assume that the charge carrier has an equal chance of ending up in either one of the 3 states.

If the charge carrier ends up in the ground state, it will remain there during the waiting stage. If the charge carrier is in one of the excited states, it might relax to a lower-energy state.

In the measurement stage, the energy level of the states is moved up by applying a voltage pulse. If the charge carrier is in the ground state, it may tunnel out. If it is in the first excited state, it may either tunnel out or relax to the ground state, and if it is in the second excited state, it may tunnel out, relax to the ground state, or relax to the first excited state. If the dot is empty at any point during the measurement stage, another charge carrier from the leads may fill either one of the 3 states, however, this time with different probabilities.

In the unloading stage, the energy level of the states is moved up way above the electrochemical potential of the leads, which causes the charge carrier to tunnel out from the quantum dot.

Probability of tunneling

All tunneling processes mentioned in the section above are probabilistic and described through tunneling rates. In the loading stage, we use tunneling rate $3\Gamma_{\text{in},0}$, because the electron can tunnel into either one of the 3 states. In the unloading stage, we use tunneling rate $\Gamma_{\text{out},0}$. In the simulation, we used the value (taken from the experiments) $\Gamma_{\text{in},0}$=$\Gamma_{\text{out},0} = 15\,\text{Hz}$.

In the measurement stage, the tunneling rate can be calculated using Fermi-Dirac distribution:

$$\Gamma_{\text{in/out}} = \frac{\Gamma_{\text{in/out},0}}{1 + e^{-\frac{E}{k_\text{B}T}}}, \tag{1}$$

where T is temperature (in the simulation we used value $T = 25\,\text{mK}$), k_B is the Boltzmann constant and E is the energy of the given state, assuming that the electrochemical potential of the leads corresponds to $E = 0$. We can notice that if the energy level of the given state is a bit below the electrochemical potential of the leads, the tunneling rate Γ_{in} is much larger than Γ_{out} and therefore it is more probable for a charge carrier to tunnel in than the opposite situation.

To calculate the probability of a charge carrier tunneling either in or out of the dot, we need to use Bayes' theorem. It states that the conditional probability of an event A happening while event B is true can be calculated using the following equation:

$$P(A|B) = \frac{P(B|A) \cdot P(A)}{P(B)}. \tag{2}$$

We can apply this theorem to calculate the probability of the dot getting occupied in time interval $[t, t + \Delta t]$, provided that the dot has been empty before that, in time interval $[0, t]$:

$$P(\text{dot got occupied in [t, t + \Delta t]|dot was empty in [0, t]}) =$$

$$= \frac{P(\text{dot got occupied in [t, t + \Delta t]}) \cdot P(\text{dot was empty in [0, t]|dot got occupied in [t, t + \Delta t]})}{P(\text{dot was empty in [0, t]})}$$

$$= \frac{\int_t^{t+\Delta t} \Gamma e^{-\Gamma t}\,dt}{1 - \int_0^t \Gamma e^{-\Gamma t}\,dt} = \frac{e^{-\Gamma t} - e^{-\Gamma(t+\Delta t)}}{e^{-\Gamma t}} = 1 - e^{-\Gamma \Delta t}. \tag{3}$$

In the calculation, we used the fact that the probability density of a tunneling event at a time t is given by

$$p(t)\,dt = \Gamma e^{-\Gamma t} dt. \tag{4}$$

In our simulation, we discretized the whole readout process, with a time step $t_{\mathrm{step}} = 0.0005\,\mathrm{s}$. In each step, we generate a random number from 0 to 1 and calculate the probability given by equation (3). If the randomly generated number is smaller than the calculated probability, the given tunneling event occurs.

F. CHARGE STABILITY DIAGRAM

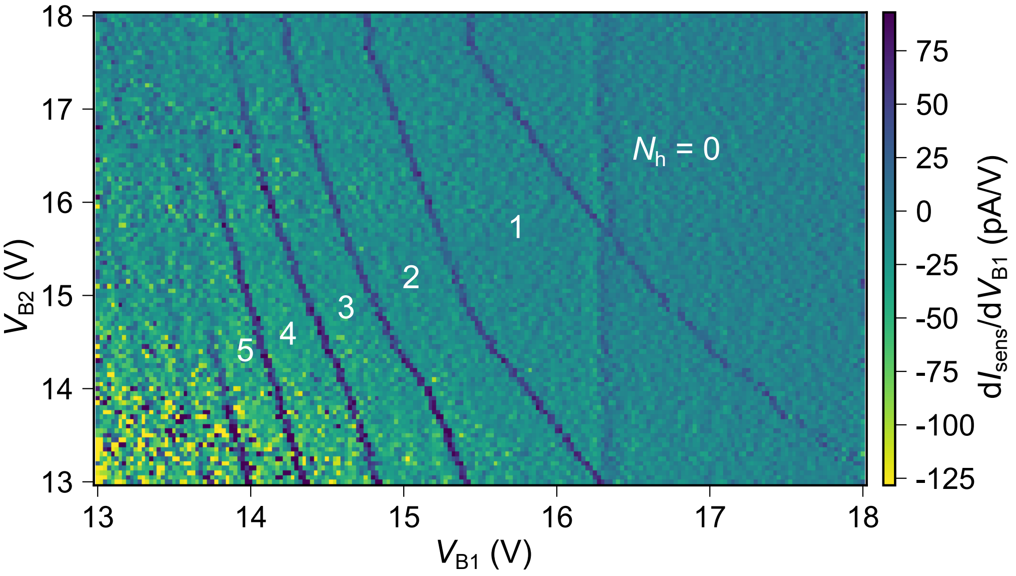

FIG. 5. **Barrier-barrier charge stability diagram.** Additional charge stability diagram. Sensor current derivative as a function of the barrier gate voltages. N_h indicates the number of hole carriers inside the QD.

[1] H. Duprez, S. Cances, A. Omahen, M. Masseroni, M. J. Ruckriegel, C. Adam, C. Tong, J. Gerber, R. Garreis, W. Huang, L. Gächter, T. Taniguchi, K. Watanabe, T. Ihn, and K. Ensslin, Spectroscopy of a single-carrier bilayer graphene quantum dot from time-resolved charge detection, 10.48550/ARXIV.2311.12949 (2023).

[2] A. Knothe, L. I. Glazman, and V. I. Fal'ko, Tunneling theory for a bilayer graphene quantum dot's single- and two-electron states, New Journal of Physics **24**, 043003 (2022).

[3] A. Kurzmann, M. Eich, H. Overweg, M. Mangold, F. Herman, P. Rickhaus, R. Pisoni, Y. Lee, R. Garreis, C. Tong, K. Watanabe, T. Taniguchi, K. Ensslin, and T. Ihn, Excited states in bilayer graphene quantum dots, Phys. Rev. Lett. **123**, 026803 (2019).

[4] J. R. Petta, A. C. Johnson, J. M. Taylor, E. A. Laird, A. Yacoby, M. D. Lukin, C. M. Marcus, M. P. Hanson, and A. C. Gossard, Coherent manipulation of coupled electron spins in semiconductor quantum dots, Science **309**, 2180 (2005).

[5] K. Flensberg and C. M. Marcus, Bends in nanotubes allow electric spin control and coupling, Phys. Rev. B **81**, 195418 (2010).

[6] E. A. Laird, F. Pei, and L. P. Kouwenhoven, A valley–spin qubit in a carbon nanotube, Nature Nanotechnology **8**, 565 (2013).

[7] G. Burkard, T. D. Ladd, A. Pan, J. M. Nichol, and J. R. Petta, Semiconductor spin qubits, Rev. Mod. Phys. **95**, 025003 (2023).

[8] C. Tong, R. Garreis, A. Knothe, M. Eich, A. Sacchi, K. Watanabe, T. Taniguchi, V. Fal'ko, T. Ihn, K. Ensslin, and A. Kurzmann, Tunable valley splitting and bipolar operation in graphene quantum dots, Nano Letters **21**, 1068 (2021).
